# Post-Operative Functional Outcomes in Early Age Onset Rectal Cancer

**DOI:** 10.3389/fonc.2022.868359

**Published:** 2022-05-30

**Authors:** REACCT Collaborative, Lauren V. O’Connell

**Affiliations:** Centre for Colorectal Disease, St Vincent's University Hospital, Dublin, Ireland

**Keywords:** functional outcome, young rectal cancer, patient reported outcome (PROM), rectal cancer, early onset rectal cancer

## Abstract

**Background:**

Impairment of bowel, urogenital and fertility-related function in patients treated for rectal cancer is common. While the rate of rectal cancer in the young (<50 years) is rising, there is little data on functional outcomes in this group.

**Methods:**

The REACCT international collaborative database was reviewed and data on eligible patients analysed. Inclusion criteria comprised patients with a histologically confirmed rectal cancer, <50 years of age at time of diagnosis and with documented follow-up including functional outcomes.

**Results:**

A total of 1428 (n=1428) patients met the eligibility criteria and were included in the final analysis. Metastatic disease was present at diagnosis in 13%. Of these, 40% received neoadjuvant therapy and 50% adjuvant chemotherapy. The incidence of post-operative major morbidity was 10%. A defunctioning stoma was placed for 621 patients (43%); 534 of these proceeded to elective restoration of bowel continuity. The median follow-up time was 42 months. Of this cohort, a total of 415 (29%) reported persistent impairment of functional outcomes, the most frequent of which was bowel dysfunction (16%), followed by bladder dysfunction (7%), sexual dysfunction (4.5%) and infertility (1%).

**Conclusion:**

A substantial proportion of patients with early-onset rectal cancer who undergo surgery report persistent impairment of functional status. Patients should be involved in the discussion regarding their treatment options and potential impact on quality of life. Functional outcomes should be routinely recorded as part of follow up alongside oncological parameters.

## Introduction

While globally colorectal cancer incidence rates are overall predominantly stable or declining, a slew of data suggests that in Westernised countries incidence in the younger population (<50 years) is markedly increasing (1–8). Although there is no agreed definition of ‘young-onset’ colorectal cancer, most literature includes those diagnosed at ≤ 50 years of age (9–11). A proportion of these are due to inherited colorectal cancer syndromes (e.g. Lynch syndrome, familial adenomatous polyposis etc.); however, the majority are sporadic (9). Colorectal cancer in the young demonstrates some distinct features comparative to the disease which occurs in the older age group. The vast majority of the increase in incidence is accounted for by left-sided tumors, with little-no increase in right-sided neoplasms (9, 10, 12). Compared to the older age group, a greater proportion of young rectal cancers present symptomatically, rather than being detected incidentally or by screening. Despite this, the interval from onset of symptoms to diagnosis is on average six months greater for the young cohort than in older adults (13, 14). As such these patients tend to present with a more advanced stage at diagnosis, with 71% diagnosed at stage III or IV (15, 16). In addition, high risk features such as signet-ring mucinous histology and poor differentiation are more frequently seen. However, these factors do not confer a worse prognosis, with stage-adjusted cancer-specific survival similar or better than older patients (10, 17–19). This may reflect more aggressive treatment strategies. Young patients with rectal cancer are more likely than older patients to undergo systemic therapy and complex surgical interventions for equivalent stage disease (19–21).

Functional impairment after treatment for rectal cancer is unfortunately common. With respect to low anterior resection syndrome (LARS), the reported incidence of major symptomatology after curative restorative anterior resection varies from 37 to up to 90% of patients (22–26). The presence of a stoma, whether permanent or temporary, has negative effects on body image – which may persist even after restoration of bowel continuity – and may delay the ability of young patients to return to work (27). Urogenital symptoms are less frequently discussed by healthcare providers but are also common (28, 29).

A diagnosis of rectal cancer in the young carries several special considerations. The increased frequency of adverse pathological features warrants aggressive management strategies to ensure optimal oncological outcomes. Nevertheless, long-term health-related quality of life (HRQOL) is also of critical importance. Particular consideration should be given to preservation of a functional sphincter complex, avoidance of a permanent stoma and autonomic nerve sparing. Other special considerations include fertility and the potential impact of pelvic radiotherapy and surgery, especially in female patients of child-bearing age. The importance of measuring functional outcomes after treatment for rectal cancer in addition to post-operative morbidity and oncological outcomes is increasingly recognized and reported. There is a paucity of data examining functional impairment after rectal cancer treatment in young-onset patients (30). This study evaluated gastrointestinal, genitourinary and fertility concerns in a large cohort of young-onset rectal cancer patients.

## Methods

### Study Participants

An international multicentre observational study to assess the functional outcomes of patients diagnosed with early age onset rectal cancer was performed. Inclusion criteria were adults aged between 18 and 49 years with a histologically confirmed diagnosis of rectal cancer, with known post-operative bowel, bladder or sexual functional status.

### Data Collection and Analysis

Patients who fulfilled the inclusion criteria of the study were selected from the REACCT Collaborative database. All participating institutions are tertiary referral units with specialist expertise in CRC. Ethical approval was sought at an individual institutional level. Collected data included baseline patient demographics, clinical, stage, surgical, and treatment data, and functional outcomes. A microscopically clear resection (R0) was defined by a tumor-free resection margin of at least 1 mm. Bowel dysfunction was defined as frequency, urgency, clustering or incontinence. Bladder dysfunction was defined as voiding difficulties or need for catheterization. Comprehensive investigation was required for a diagnosis of infertility. Basic descriptive statistics were calculated.

## Results

A total of 1,428 patients were included in the study. The median (range) age was 42 (18–49) years and 816 (57%) were male. Median (range) BMI was 22 (13–50). The majority (86%) had non-metastatic disease. Baseline demographics are summarised in **Table 1**.

**Table 1:** Baseline demographics and clinical characteristics.

	N = 1428
**Median age (range**	42 (18-49)
**Male**	816 (57%)
**Median BMI (range)**	22 (13-50)
**cTNM stage**	
I-III	1244
IV	184
**Distance to anal verge; Median (range)**	8 (0-15)
**Neoadjuvant therapy**	572
**Type of neoadjuvant therapy**	
Chemoradiotherapy	487
Radiotherapy only	12
**pTNM stage**	
I	
II	374
III	516
IV	206
**R0 resection**	1212

All patients underwent surgical intervention with 97% (n=1395) having an elective procedure. Neoadjuvant therapy was administered to 39% patients. The median (range) interval to surgery was 8 (4-67) weeks. The most commonly performed operation was a low anterior resection (LAR) whilst 17% underwent an APR and 19% had another procedure such as local excision (LE). Defunctioning stomas were placed in two-thirds of those undergoing LAR. Adjuvant chemotherapy was given to 51% (n=724). Operative details are summarized in **Table 2**.

**Table 2:** Operative details and post-operative morbidity.

	N = 1428
**Elective surgery**	1395 (97%)
**Mechanical bowel preparation**	1000
**Median interval to surgery; weeks (range)**	8 (4-67)
**Type of operation**	
Abdominoperineal resection	244
Low anterior resection	907 (64%)
Other	277
**Operative approach**	
Laparoscopic	576
Open	822
Robotic	30
**Defunctioning ileostomy**	621 (43%)
**In-hospital mortality**	52
**Major post-operative complication (Clavien Dindo 3-4)**	143
**Ileostomy reversal**	534

The rate of major post-operative morbidity within 30 days was 9.6%, with ileus the most commonly reported post-operative morbidity. The median follow-up was 42 months (range 1-180). The most common functional impairment related to the gastrointestinal tract. Symptoms of bowel dysfunction such as frequency, urgency, clustering or incontinence were present in 17% (n=235). Urinary dysfunction (e.g. voiding difficulties or the need for intermittent self-catheterization) was reported by 7% (n=100) whilst 6% (n=65) experienced symptoms of sexual dysfunction. Only fifteen patients reported fertility-related problems.

## Discussion

In general, some degree of functional impairment post treatment for rectal cancer is extremely common. With respect to bowel dysfunction, as outlined in the Introduction, rates of up to 90% have been reported. These symptoms may persist well beyond the initial post-operative phase, with as many as 71-74% patients reporting some degree of faecal incontinence and rectal evacuation difficulties at 15 years post-surgery (25, 31, 32). The prevalence of major LARS at long-term follow up is high, with data from a recent meta-analysis reporting its frequency at 41% (33, 34). The need for a defunctioning stoma may also be associated with post-operative LARS (35, 36). Furthermore, a longer interval from stoma formation to restoration of bowel continuity may be associated with worse final bowel function, particularly with respect to urgency and continence (35, 37). In the present study of functional outcomes in young patients with rectal cancer, 29% reported bowel, bladder and/or sexual dysfunction.

Impairment of bladder and sexual function in patients with rectal cancer are also common, although these are less frequently reported on than bowel dysfunction, especially in female patients (38, 39). These effects are primarily due to division of the pelvic autonomic nerves during surgery, although some symptoms may also be a sequela of pelvic radiotherapy. Aside from impact on bowel function, presence of a stoma is associated with worse sexual function, negative body image and a delay in return to work (27, 40). Rates of urinary dysfunction post treatment range from 58-77% of patients reporting urgency, 46% voiding difficulty and 20-63% urinary incontinence (39, 41). Rectal cancer patients also report more sexual dysfunction symptoms and body image issues comparative to those with more proximal neoplasms. Up to 54% of male rectal cancer survivors report erectile dysfunction in contrast to 25% of those with colonic cancer (42). In women, dyspareunia occurs in 26-53% and vaginal dryness in up to 75% (39, 43). Incidence of sexual dysfunction in both males and females appears to be associated those undergoing with APR and who received NCRT (38, 39, 44, 45).

The data above pertain to patients of all ages. Despite the rapid acceleration in rectal cancer incidence in the younger population, there is scant literature specifically examining post-operative functional outcomes in young-onset rectal cancer patients. Nevertheless, this cohort has specific needs and considerations. Data from the QoLiRECT study suggests that younger patients are disproportionately affected by LARS (34). Aside from impact on bowel function, presence of a stoma is associated with worse sexual function, negative body image and a delay in return to work (27, 40). For premenopausal female patients the effect of treatment on fertility must also be borne in mind, primarily due to pelvic radiotherapy but also potentially from adhesions post pelvic surgery and the effect of chemotherapeutic drugs such as oxaliplatin (46–49).

To our knowledge, this is the first large-scale series reporting the incidence of bowel, urogenital and fertility-related dysfunction in a cohort of young-onset rectal cancer patients. Although not approaching the rates reported in some of the literature described above, nevertheless a substantial minority of patients reported impairment of at least one functional domain. In line with the existing body of literature, bowel dysfunction was the most prevalent functional impairment, followed by bladder and sexual dysfunction. There are several acknowledged contributing factors which increase the likelihood of functional impairment post-operatively. These include tumor height – thus influencing the surgical procedure undertaken and feasibility of sphincter preservation -, administration of NCRT, TME versus organ sparing approaches, and occurrence of post-operative morbidity, in particular anastomotic leak.

Many of the factors described above which affect functional impairment post-treatment are non-modifiable. Therefore, thorough pre-operative counselling on the potential outcomes to ensure informed consent and engaging in a shared decision-making process with these patients on the risks and benefits of individual treatment options is critical. This process may be assisted by utilization of aids such as the pre-operative LARS score (POLARS) tool, a nomogram which inputs clinico-pathological characteristics including patient age, tumor height, administration of NCRT, planned total or partial mesorectal excision and plan for a defunctioning stoma in order to predict the likelihood of post-operative bowel dysfunction (50). Models also exist to predict the likelihood of permanent stoma at 2 years post-operatively and may similarly be used to assist in providing informed consent and facilitate shared decision-making between patients and healthcare providers (51). Patients of reproductive age should be specifically counselled on and referred for fertility preservation options prior to commencement of treatment (20, 52, 53).

Consideration should be given to organ or sphincter-preserving and nerve-sparing approaches where feasible without threatening oncological safety. Data from the International Watch and Wait Database suggests that a strategy of non-operative management in young-onset patients with a cCR is not associated with worse oncological outcomes (54). For low-risk early rectal cancers, organ-sparing local excision in the form of transanal endoscopic microsurgery (TEM) or transanal minimally invasive surgery (TAMIS) with or without NCRT may be adequate and avoid the functional morbidity associated with total mesorectal excision (55–58). In patients with locally advanced rectal cancer, total neoadjuvant treatment (TNT) in the form of radiotherapy with either induction or consolidation chemotherapy appears to result in increased rates of pCR, reduced toxicity comparative to adjuvant chemotherapy and improved survival (59–62). For patients requiring TME, there is some evidence in the literature to suggest that utilization of a robotic-assisted approach over laparoscopic may facilitate sparing of the pelvic autonomic nerves and thus reduce the incidence of post-operative bladder and sexual dysfunction (63–67). However, further data will be required to definitively confirm this. In this series, only 2% of procedures were undertaken robotically, although this proportion is likely to increase over time with the burgeoning uptake of the technology.

The decision whether to place a defunctioning stoma should be carefully weighed against the negative impacts on sexual function, ability to return to work and body image (20, 27, 40, 42). Early recognition and prompt treatment of anastomotic leak is important to minimize adverse effects on long-term bowel function (68–70). Restoration of bowel continuity where a diverting stoma has been placed should be a priority, as a long interval to reversal is associated with dysfunction (37).

In the immediate post-operative period, the major consideration should be early recognition and prompt treatment of morbidity, specifically anastomotic leak, to minimize the recognized adverse effect of this complication on long-term bowel function (68–70). After the immediate post-operative period, restoration of bowel continuity where a diverting stoma has been placed should be a priority, as a longer interval to reversal of a defunctioning stoma is associated with a higher incidence of long-term faecal incontinence (37). At long-term follow-up, functional outcomes should be routinely recorded alongside oncological parameters.

In conclusion, future studies focusing on patient-reported as well as oncological outcomes in young rectal cancer patients will result in improved characterization of functional outcomes in this group. Enhanced functional outcomes *via* organ preservation, sphincter preservation and other approaches will have consequent positive effects on quality of life.

## Data Availability Statement

The raw data supporting the conclusions of this article will be made available by the authors, without undue reservation.

## Ethics Statement

The studies involving human participants were reviewed and approved by Research Ethics Committee, St Vincent’s University Hospital. Written informed consent for participation was not required for this study in accordance with the national legislation and the institutional requirements.

## REACCT Collaborative

Members of the REACCT Collaborative are:

Lauren V. O’Connell, Alexandra M. Zaborowski, Ahmed Abdile. Michel Adamina, Felix Aigner, Laura d’Allens, Caterina Allmer, Andrea Álvarez, Rocio Anula, Mihailo Andric, Sam Atallah Simon Bach, Miklosh Bala, Marie Barussaud, Augustinas Bausys, Andrew Beggs, Felipe Bellolio, Melissa-Rose Bennett, Vicki Bevan, Sebastiano Biondo, Gabriele Bislenghi, Marc Bludau, Carl Brown, Christiane Bruns, Daniel D. Buchanan, Pamela Buchwald, Jacobus W.A. Burger, Nikita Burlov, Michela Campanelli, Maylis Capdepont, Michele Carvello, Hwee-Hoon Chew, Dimitri Christoforidis, David Clark, Marta Climent, Rowan Collinson, Kyle G. Cologne, Tomas Contreras, Roland Croner, Ian R. Daniels, Giovanni Dapri, Justin Davies, Paolo Delrio, Quentin Denost, Michael Deutsch, Andre Dias, André D’Hoore, Evgeniy Drozdov, Daniel Duek, Malcolm Dunlop, Adam Dziki, Aleksandra Edmundson, Sergey Efetov, Alaa El-Hussuna, Brodie Elliott, Sameh Emile, Eloy Espin-Basany, Martyn Evans, Seraina Faes, Omar Faiz, Nuno Figueiredo, Fergal Fleming, Caterina Foppa, George Fowler, Matteo Frasson, Tim Forgan, Frank Frizelle, Shamil Gadaev, Jose Gellona, Tamara Glyn, Barisic Goran, Emma Greenwood, Marianne G. Guren, Stephanie Guillon, Ida Gutlic, Dieter Hahnloser, Heather Hampel, Ann Hanly, Hirotoshi Hasegawa, Lene Hjerrild Iversen, Andrew Hill, James Hill, Jiri Hoch, Roel Hompes, Luis Hurtado, Fabiano Iaquinandi, Ugne Imbrasaite, Rumana Islam, Mehrenah D Jafari, Andrea Jiménez Salido, Marta Jiménez-Toscano, Yukihide Kanemitsu, Aleksei Karachun, Ahmer A. Karimuddin, Deborah S. Keller, Justin Kelly, Rory Kennelly, Gleb Khrykov, Petr Kocian, Cherry Koh, Neils Kok, Katrina A. Knight, Joep Knol, Christos Kontovounisios, Hartwig Korner, Zoran Krivokapic, Irmgard Kronberger, Hidde Maarten Kroon, Marius Kryzauskas, Said Kural, Miranda Kusters, Zaher Lakkis, Timur Lankov, David Larson, György Lázár, Kai-Yin Lee, Suk Hwan Lee, Jérémie H. Lefèvre, Anna Lepisto, Christopher Lieu, Lynette Loi, Craig Lynch, Helene Maillou-Martinaud, Annalisa Maroli, Sean T. Martin, Anna Martling, Klaus E. Matzel, Julio Mayol, Frank McDermott, Guillaume Meurette, Monica Millan, Martin Mitteregger, Andrei Moiseenko, John RT. Monson, Stefan Morarasu, Konosuke Moritani, Gabriela Möslein, Martino Munini, Caio Nahas, Sergio Nahas, Ionut Negoi, Anastasia Novikova, Misael Ocares, Koji Okabayashi, Alexandra Olkina, Luis Oñate-Ocaña, Jaime Otero, Cihan Ozen, Ugo Pace, Guilherme Pagin São Julião, Lidiia Panaiotti, Yves Panis, Demetris Papamichael, Swati Patel, Juan Carlos Patrón Uriburu, Sze-Lin Peng, Miguel Pera, Rodrigo O. Perez, Alexei Petrov, Frank Pfeffer, Terry P. Phang, Tomas Poskus, Heather Pringle, David Proud, Ivana Raguz, Nuno Rama, Shahnawaz Rasheed, Manoj J. Raval, Daniela Rega, Christoph Reissfelder, Juan Carlos Reyes Meneses, Frederic Ris, Stefan Riss, Homero Rodriguez-Zentner, Campbell S Roxburgh, Avanish Saklani, Tarik Sammour, Deborah Saraste, Martin Schneider, Ryo Seishima, Aleksandar Sekulic, Toni Seppala, Kieran Sheahan, Alexandra Shlomina, Guiseppe Sica, Tongplaew Singnomklao, Leandro Siragusa, Neil Smart, Alejandro Solis-Peña, Antonino Spinelli, Roxane D. Staiger, Michael J. Stamos, Scott Steele, Ker-Kan Tan, Pieter J Tanis, Paris Tekkis, Biniam Teklay, Sabrina Tengku, Petr Tsarkov, Matthias Turina, Alexis Ulrich, Bruna B. Vailati, Meike van Harten, Cornelis Verhoef, Satish Warrier, Steven Wexner, Benjamin A. Weinberg, Cameron Wells, Albert Wolthuis, Evangelos Xynos, Nancy You, Alexander Zakharenko, Justino Zeballos, Youzhi Zhou, Des C. Winter.

## Author Contributions

LO’C: drafting of manuscript. DW: conceptualization of study, editing of manuscript and confirmation of final version for submission. AMZ: drafting and editing of manuscript, approval of final version for submission. All other authors - data collection and submission, statistical analysis. All authors contributed to the article and approved the submitted version.

## Conflict of Interest

The authors declare that the research was conducted in the absence of any commercial or financial relationships that could be construed as a potential conflict of interest.

## Publisher’s Note

All claims expressed in this article are solely those of the authors and do not necessarily represent those of their affiliated organizations, or those of the publisher, the editors and the reviewers. Any product that may be evaluated in this article, or claim that may be made by its manufacturer, is not guaranteed or endorsed by the publisher.
